# Bone fragility in a post-intestinal transplant patient with mesenteric heterotopic ossification

**DOI:** 10.1093/jbmrpl/ziag082

**Published:** 2026-05-12

**Authors:** Husam Hamshary, Dania S Bacha, Leila Z Khan

**Affiliations:** Department of Medicine, An-Najah National University, Nablus, P400, Palestine; Department of Endocrinology, University of Pennsylvania, Philadelphia, PA 19104, United States; Department of Endocrinology, Calcium/Parathyroid Center, Cleveland Clinic, Cleveland, OH 44195, United States

**Keywords:** heterotopic ossification, mesenteric ossification, osteoporosis, intestinal transplantation, vertebral fractures, denosumab, zoledronic acid, BMD

## Abstract

Heterotopic ossification (HO) is the aberrant formation of mature lamellar bone in extraskeletal tissues, most commonly following trauma or surgery. Mesenteric involvement is rare and often discovered incidentally. We report the case of a 44-yr-old man with mesenteric HO in the setting of multiple emergent abdominal surgeries after a motor vehicle accident. Histopathology confirmed bone with cartilage and BM within the mesentery. He subsequently required an isolated small-intestinal transplantation for short gut syndrome. Following transplantation, the patient developed multiple vertebral compression fractures in the setting of bone fragility due to chronic antirejection medications and high-dose steroid use. He received more than a decade of treatment with zoledronic acid, with stabilization of BMD and no additional fractures. He was later transitioned to denosumab. This case underscores the rarity of HO occurring in the mesentery and highlights the challenges of managing bone fragility in high-risk post-intestinal transplant patients. This case highlights the importance of monitoring and managing bone fragility in patients undergoing intestinal transplantation and long-term immunosuppressive therapy.

## Introduction

Heterotopic ossification (HO) is the pathological formation of extra mature lamellar bone in soft tissues where bone does not normally exist, such as muscle and soft tissues, and rarely the mesentery.^1^ It was first noted in the seventeenth century in France.^2^ It is most associated with musculoskeletal trauma, central nervous system injuries, and orthopedic procedures. The condition poses significant management and clinical challenges, especially when occurring alongside osteoporosis.^3^

Heterotopic ossification represents a complex biological response to local and systemic inflammation. The process begins with the inappropriate differentiation of mesenchymal stem cells into osteoblasts under the influence of osteogenic factors like BMPs and TGF-β. This can be exacerbated by local hypoxia, immobility, and immune dysregulation.^4^

In our case, we describe a patient with mesenteric HO. After the lesions were removed, he required an intestinal transplant for short gut syndrome and, unfortunately developed subsequent vertebral fractures secondary to his immunosuppressant regimen. BMD was nicely stabilized with bisphosphonate and denosumab treatments. This case highlights the rarity of HO occurring in the mesentery and underscores the importance of monitoring bone health in patients undergoing intestinal transplantation and receiving long-term immunosuppressive therapy.

## Case presentation

**Figure 1:**
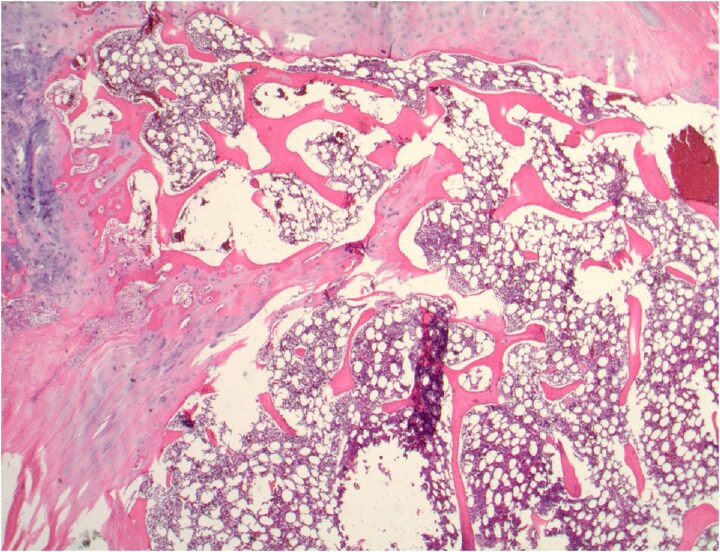
Mesentery contains cartilage, bone, and fatty BM (image courtesy of Dr Ilyssa O. Gordon).

A 44-yr-old man sustained severe injuries in a motor vehicle accident in 2003, requiring multiple abdominal surgeries; his post-trauma course was complicated by small bowel obstruction and pancreatitis. In 2016, the patient required an exploratory laparotomy for perforated diverticulitis, complicated by recurrent gastrointestinal bleeding, sepsis, and peritonitis. During this surgery, multiple fragments of bone containing cartilage and BM were noted throughout his mesentery, contributing to local inflammation and recurrent diverticulitis. The presence of bone with cartilage was subsequently confirmed by pathological specimens (Figure 1). Although exact dimensions were not recorded, this clinical presentation was felt to be consistent with HO in the abdominal cavity by the surgical team. The heterotopic mesenteric ossification was resected through multiple bowel resections, preserving only the duodenum and rectal stump, with the creation of an end jejunostomy. This extensive resection ultimately resulted in short bowel syndrome (SBS). The patient later elected to undergo an isolated small intestinal transplant to improve the quality of life associated with his SBS.

Baseline DXA scan in 2016 before intestinal transplant revealed lower than expected scores with BMD of 0.788 g/cm^2^ at the left femoral neck (*Z* score −1.7), and 0.761 g/cm^2^ at the left TH (*Z* score of −2.1). Following intestinal transplantation in 2017, he was initiated on tacrolimus and hydrocortisone for immunosuppression. Within months, he developed sudden severe mid-back pain while riding an elevator, and imaging confirmed acute vertebral fractures at T8 and T9. The patient’s bone fragility was attributed to high-dose corticosteroid and immunosuppressant therapy, with other potential causes excluded. There was no family history of bone loss or osteoporosis. Relevant laboratory investigations, including serum calcium, phosphorus, PTH, creatinine, and vitamin D levels, were reviewed and summarized in Table 1. Repeat DXA scan in 2017 showed a decline with BMD of the left femoral neck of 0.748 g/cm^2^ (*Z* score of −2.1) with a −5.1% decline in left femur density. The density of the total left hip was stable at 0.726 g/cm^2^ (*Z* score of −2.3). Over the following year, he sustained multiple additional vertebral fractures (T12) requiring kyphoplasty. These injuries resulted in progressive kyphosis, a loss of ~3 inches in height, and a significant limitation in mobility.

**Table 1:** Laboratory investigations.

**Date**	**Calcium (8.6-10 mg/dL)**	**Albumin (3.9-4.9 g/dL)**	**PTH (15-65 pg/mL)**	**Phosphorus (2.7-4.8 mg/dL)**	**Creatinine (0.73-1.22 mg/dL)**	**Vitamin D 25 (ng/mL)**
**11/5/2016**	9.7	3.8	43	2.9	0.89	41.4
**10/3/2017**	9.4	3.8	34	3.8	1.74	38.5
**10/1/2018**	9.5	3.7	61	3.3	1.38	67.7
**9/15/2019**	10.1	3.7	63	3.9	1.23	65.8
**10/5/2020**	9.7	4.0	—	4.17	1.47	57.6
**4/23/2021**	9.5	3.7	62	2.9	1.35	75.8
**5/10/2022**	9.6	4.0	—	2.5 (L)	1.24 (H)	53.1
**6/4/2023**	9.6	3.8	62	3.2	1.17	53.8
**11/13/2024**	9.4	3.4	—	3.0	0.99	53
**7/13/2025**	9.7	4.0	67	3.2		

Our patient received antiresorptive therapy with zoledronic acid for 10 yr, and then was transitioned to denosumab. BMD has remained stable on serial DXA scans, with no new fractures reported, although a new machine was used, so a direct comparison is not possible (Table 2).

**Table 2:** BMD.

**Date**	**Total left hip BMD (g/cm** ^2^ **), *Z*-score**	**Left FN BMD (g/cm** ^2^ **), *Z*-score**		
**November 30, 2016**	0.761, *Z* −2.1	0.788, *Z* −1.7		First dose of bisphosphonate
**October 11, 2017**	0.726, *Z* −2.3	0.748, *Z* −2.1	S/P isolated intestinal transplant 4/13/17	Second dose of bisphosphonate
**June 13, 2023**	0.772, *Z* −1.9	0.834^a^, *Z* −1.2		Seventh dose of bisphosphonate
**July 06, 2024**	0.788, *Z* −1.8	0.851, *Z* −1.0		Initiation of denosumab
**July 07, 2025**	0.805, *Z* −1.7	0.877, *Z* −0.8		Ongoing denosumab use

This table summarizes serial BMD measurements.

DXA systems used

• 2016-2017, 2023: A50 Lunar Prodigy Advance (S/N: PA + 300 680)

• 2024-2025: A21 Lunar Prodigy (S/N: PA + 512 469)

DXA BMD over time

Total left hip and left FN results

DXA systems used

• 2016-2023: A50 Lunar Prodigy Advance (S/N: PA + 300 680)

2024-2025: A21 Lunar Prodigy (S/N: PA + 512 469)

a Between 2017 and 2023, the left hip neck BMD increased by +5.2%, which exceeds typical least significant change thresholds and is therefore a significant increase.

## Discussion

Heterotopic ossification is a complex pathological process involving the aberrant formation of bone in non-skeletal tissues, with lamellar bone forming in non-ossified soft tissue. It is characterized by the formation of mature, extraskeletal bone in soft tissues, including muscle, fascia, tendon, ligament, subcutis, and skin.^5^ Histologically, HO is characterized by a zonal pattern with peripheral hypercellular areas of spindle cells transitioning into central zones containing mature bone.^4^ The mechanism of HO involves inappropriate differentiation of local mesenchymal stem cells into bone-forming osteoblasts. This is driven by BMPs, TGF-β, VEGF, and pro-inflammatory cytokines such as IL-1β and IL-6, particularly in the setting of trauma and inflammation.^5^ Additionally, hypoxia and mechanical stress play key roles in upregulating osteoinductive signaling pathways.^6^

The clinical presentation of HO varies by location. It may be asymptomatic or cause localized pain, swelling, warmth, and a restricted range of motion due to soft tissue contractures.^7^ Limitations in range of motion can lead to further complications such as disuse osteoporosis, an inability to maintain personal hygiene, and skin maceration.^8^ In the spine or paraspinal areas, it may cause nerve compression or functional impairment. There can be intractable pain and eventual fractures during transfers or falls; complications of HO can even result in death.^9^

The incidence of HO is highest following orthopedic procedures such as TH arthroplasty, with reported rates between 20% and 53%, particularly in male patients or those with previous trauma.^3^ Heterotopic ossification is also seen in 20%-25% of burn patients, particularly those with prolonged ICU stays or delayed wound closure.^4^ The most affected sites include the hips, knees, shoulders, and elbows, with paraspinal and mesenteric involvement being rare but clinically significant.^3^ Heterotopic ossification is associated with several risk factors, including male sex, repetitive surgeries, spinal cord injury, surgery or trauma, traumatic brain injury, severe burns, immobility, genetic predisposition (eg, fibrodysplasia ossificans progressiva), and the use of bone grafts or prostheses.^3^^,^^4^

The presence of mesenteric HO following bowel resection has been documented and is believed to occur in the setting of chronic inflammation and tissue ischemia.^3^ Diagnosis relies on clinical suspicion and imaging. X-rays may show mature ossifications 3-6 wk after onset, while CT scans provide better anatomical detail. Bone scintigraphy can detect metabolically active HO. MRI may help differentiate HO from abscesses or neoplasms in unclear cases. Histopathology remains the gold standard and shows a classic zonal pattern, with central mature bone and peripheral cellular fibroblastic tissue.^4^ Treatment strategies for HO include surgical excision in symptomatic cases, along with pharmacologic prophylaxis such as Nonsteroidal Anti-Inflammatory (NSAID) drugs (s), bisphosphonates, and radiotherapy.^4^ While HO is most frequently managed via NSAIDs, bisphosphonates (eg, etidronate), radiotherapy, or surgery aiming to limit ectopic bone growth, their preventive efficacy remains inconsistent.^10^

In this case, the patient’s pathology findings included mature lamellar bone and BM in mesenteric adipose tissue and were felt to clinically represent HO. It was incidentally noted. Its cause was likely multifactorial and included multiple abdominal surgeries, prolonged immobilization, and chronic inflammation acting synergistically. Our patient required immunosuppression with hydrocortisone due to an intestinal transplant. Hydrocortisone, like all steroids, where even low-dose or short-term use, causes early and significant bone loss by disrupting both bone formation and resorption, along with worsening calcium metabolism and decreasing bone vascularization.^11^ Metabolic bone disease (MBD) is a major concern in solid organ transplant recipients, including those undergoing liver, lung, heart, and kidney transplants.^12^ Research on MBD in intestinal transplant recipients is limited. In these patients, MBD is multifactorial, involving both pre- and post-transplant factors. Intestinal failure often worsens bone disease, with conditions like Crohn’s disease and nutrient malabsorption playing key roles.^13^ Additionally, glucocorticoid exposure further disrupts bone health by altering the balance between bone formation and resorption. Only one descriptive study, conducted by Resnick et al.^14^, has addressed bone health in this population, underscoring an urgent need for further investigation to improve management strategies for bone health in intestinal transplant patients. Effective prevention includes antiresorptive treatment in addition to supplements, lifestyle measures, and the evaluation of secondary causes of bone fragility. A Cochrane review of 27 randomized controlled trials found high-quality evidence that bisphosphonates reduce the risk of vertebral fractures in glucocorticoid-induced osteoporosis, with data extending to 24 mo of use, and can prevent bone loss at both the lumbar spine and femoral neck.^15^ Our patient’s progressive vertebral fractures were likely due to the effects of bone loss post-transplant.^4^ These mechanisms are thought to be due to systemic immune dysregulation and repeated surgical interventions. Our patient tolerated multiple doses of zoledronic acid well and later transitioned to denosumab, with stable BMD outcomes. The most recent DXA showed stabilization of density on this antiresorptive treatment.

## Conclusion

This case of vertebral fractures following intestinal transplantation highlights the importance of monitoring bone fragility in transplant patients. Prompt administration of osteoporosis medications in the setting of high-dose steroids is needed to preserve function and quality of life in transplant patients, particularly intestinal transplant patients.

## Data Availability

No new data were created or analyzed in this study. Data sharing is not applicable.
